# Unusual Forehead Tremor in Four Patients with Essential Tremor

**DOI:** 10.1155/2012/278140

**Published:** 2012-07-17

**Authors:** Jordi Gascón-Bayarri, Jaume Campdelacreu, Màtil Calopa, Serge Jaumà, Laura Bau, Mònica Povedano, Jordi Montero

**Affiliations:** Departament de Neurología, Hospital Universitari de Bellvitge, 08907 L'Hospitalet de Llobregat, Spain

## Abstract

Forehead tremor has only been reported in two patients with essential tremor, one with rhythmic tremor and the other with dystonic tremor. We report 4 new patients with essential tremor who present a 4–6 Hz frontal tremor registered by electromyography and unusual features like frontal tremor preceding limb tremor or unilateral involvement. Frontal tremor is present in some patients with essential tremor, sometimes preceding limb tremor. Treatment with botulinum toxin may be useful.

## 1. Introduction

Essential tremor (ET) is typically a postural or kinetic tremor of upper limbs. Other body parts may be affected, and rarely facial tremor appears. Forehead tremor has only been reported in two patients with ET [1, 2], one of them with clinical and EMG data suggesting an additional dystonic tremor. We present 4 patients with ET in whom forehead tremor was observed. 

## 2. Patients and Methods

Four patients with essential tremor in whom frontal tremor was observed were identified. An electromyographic register was obtained in 3. Informed consent was obtained.

### 2.1. Patient 1

This patient was a 78-year-old woman, with a familial history of tremor in her mother and maternal grandfather. She complained of tremor since the age of 50, initially affecting both hands, and at age 66 she also developed head tremor. The tremor increased when she held an object and prevented her from sewing. When she was first evaluated in our service she had a no-no head tremor, a moderate rest tremor of the right arm, mild bilateral postural tremor of the hands predominantly in the left and moderate bilateral kinetic tremor on finger-to-nose. She complained of a continuous rhythmic eyebrow elevation which embarrassed her. The rest of the neurological examination was normal, and cranial CT scan and blood tests ruled out secondary causes. With a diagnosis of ET, propranolol 40 mg/12 h was started. Limb tremor and eyebrow tremor partially improved. In the next visit, 5 U of botulinum toxin were injected in each frontalis, and frontal tremor almost disappeared. Two years later, a chin tremor appeared, which was also treated with 5 U of botulinum toxin on each side. Propranolol was withdrawn due to recently diagnosed pulmonary hypertension. Limb tremor worsened and primidone 125 mg/day was started, with an initially good response. Throughout the last 4 years of followup, there was a worsening of head and upper limb tremor despite increasing primidone. Frontal and chin tremor has been successfully treated with botulinum toxin injections every 4 months. 

### 2.2. Patient 2

**Figure 1:**
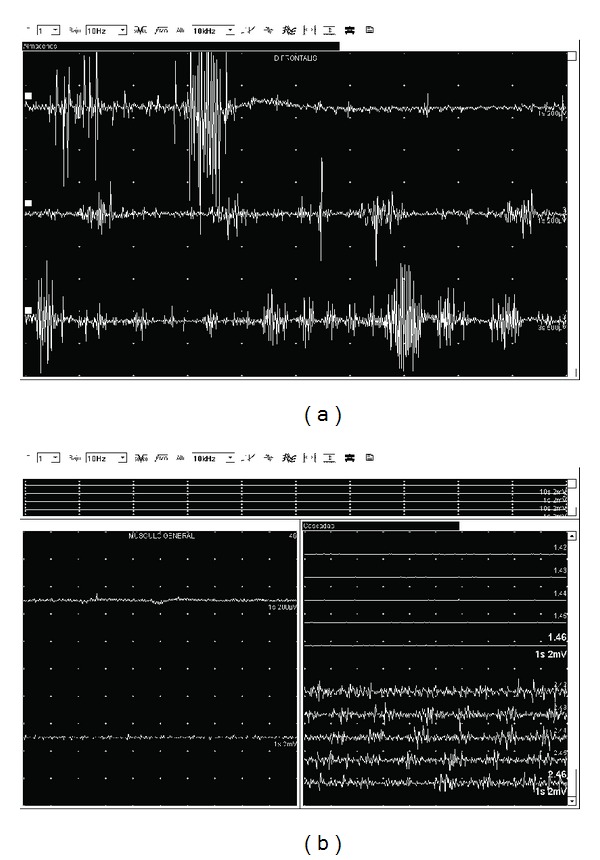
EMG recording a semirhythmic tremor in right frontalis muscle. (a) 5-6 Hz tremor (Patient 2) with patient speaking. (b) 6 Hz tremor (Patient 3) with patient speaking.

This patient was a 79-year-old woman with a history of bronchiectasis and depression treated with paroxetine and no familial history of tremor. She complained of voice tremor and oromandibular movements since 5 years ago. On examination she had a “yes-yes” head tremor, rhythmic eyebrow elevation, chin tremor, and voice tremor. She did not show limb tremor, and the rest of the examination was normal. A cranial CT scan and blood tests ruled out secondary causes. An EMG study was performed. Right frontalis muscle activity was recorded with a monopolar needle electrode using a 10 Hz low-pass filter and a 100 kHz high-pass filter. A rhythmic 5-6 Hz tremor was registered both at rest and while speaking (Figure 1(a)).

Treatment with primidone 125 mg/12h was started and dose gradually increased, but 4 years later it was stopped due to lack of efficacy. After 6 years of followup, voice tremor persists, and a postural right-hand tremor has appeared. Chin tremor has improved with 7.5–10U of botulinum toxin on each side every 4 months.

### 2.3. Patient 3

This patient was a 72-year-old man with a history of smoking, dyslipemia treated with statins, angina pectoris and duodenal ulcer, and no family history of tremor. He complained of bilateral hand tremor since one year ago, voice tremor, and right-side forehead tremor. On examination, he had a moderate bilateral upper limb postural and kinetic tremor, mild voice tremor and unilateral right frontal tremor. A cranial CT scan and blood tests ruled out secondary causes. The EMG register showed a 6Hz intermittent tremor in the right frontalis muscle (Figure 1(b)) when the patient was speaking or counting (See Supplementary material available on line at doi:10.1155/2012/278140 Video) but not at rest. He was diagnosed of ET and propranolol was started with a mild improvement of hand tremor. Voice tremor and frontal tremor persist but are not annoying for the patient. After 4 years of followup, he is clinically stable with propranolol 40 mg/12 h. 

### 2.4. Patient 4

This patient was a 78-year-old woman with a history of hypertension and asthma and no family history of tremor. She presented a bilateral upper limb action tremor, predominantly in the left hand, since 2 years ago, which made it difficult to drink from a glass of water or eat soup. On examination, she had a mild postural tremor of the right hand, moderate in the left hand, moderate intention tremor of the right hand, severe in the left hand, chin tremor, head tremor, and intermittent eyebrow tremor. The rest of the examination, blood tests, and cranial CT scan were normal. The EMG registered a 4–6 Hz rhythmic frontal tremor. She has a contraindication for propranolol and is receiving diazepam 5 mg/day prior to the neurologist's visit with subjective improvement. No other treatments have been tried yet.

## 3. Discussion

Tremor is the most common movement disorder. Classical ET is a postural and kinetic tremor with a frequency of 4–12 Hz, without evidence of dystonia and in the absence of other conditions or drugs which could cause the tremor [3]. The upper limbs are usually affected, followed by head and voice tremor [4]. Facial tremor is unusual in patients with ET [3–5]. In a series of 350 patients with ET, the localizations of tremor were hands (89.7%), head (40.9%), voice (17.7%), legs (13.7%), jaw (7.1%), face (2.9%), trunk (1.7%), and tongue (1.4%) [4]. In another review [5], the affected facial muscles were orbicularis oculi, orbicularis oris, and mentalis. In this series, facial tremor was not an isolated tremor in any case. On the contrary, it appeared in patients already affected with a severe upper limb tremor. In a population study of 175 cases of ET in Papua New Guinea [6], the authors specified that superior facial muscles (frontalis and periorbital muscles) were not affected in any case. 

We have only found two reports in the literature of patients with frontal tremor. The first was described in a patient with a history of 18 years of ET, initially involving the upper limbs and later the head [1]. This patient had a bilateral eyebrow elevation and forehead tremor when he was speaking, but not at rest or when he voluntarily tried not to raise his eyebrows. The presence of this task-specific tremor and the neurophysiological findings of low-frequency irregular tremor, cocontraction of antagonist muscles and EMG bursts suggested that the tremor was dystonic. The authors concluded that forehead tremor in a patient with ET probably indicates an additional pathophysiological process. Dystonic tremor is difficult to distinguish from action tremor in ET. Moreover, the coexistence of ET and dystonia is not unusual, and the neurophysiologic study may aid in the diagnosis [1]. The other case is mentioned in a series of 94 ET patients [2], where it is described that tremor involved cranial structures in 74.5% of cases (neck, voice, chin, tongue or face). The authors specifically highlight that one of the patients had forehead tremor, and it is shown in a video segment.

Unlike previously reported series, where cranial muscles are involved only in the presence of severe upper limb tremor, one of our patients experienced facial tremor in the absence of upper limb tremor, which manifested 6 years later. It is also interesting that one of the patients had a unilateral right frontal tremor. 

In summary, we present 4 patients with ET and frontal tremor, some of them with unusual features not previously reported like preceding limb tremor or unilateral involvement. Frontal tremor in these patients is semiregular but not exactly rhythmic in the EMG register, has a 4–6 Hz frequency and resembles the patient described by Piboolnurak et al. [1], that was considered dystonic. Patients with frontal tremor also had head tremor (2) or facial tremor in other localizations like voice (2) or chin (3).

As a practical issue, it is important to highlight the good response to botulinum toxin for frontal or chin in two of our patients, while classical treatments like propranolol or primidone had failed. 

We must point out that after the first case we have paid more attention to the possible presence of forehead tremor. The specific search for frontal tremor will possibly reveal that it is not an exceptional sign and allow future studies to better understand the pathophysiology of facial tremor and why certain muscles are more or less frequently involved. Symptomatic treatment of frontal or chin tremor with low doses of botulinum toxin should be considered as a safe and effective option in these patients.

## Supplementary Material

Video of Patient 3, a 72-year-old man with essential tremor, showing intermittent tremor in the right frontalis muscle while the patient is counting. This tremor appeared when speaking or counting but not at rest.
